# The Correlation between Core Muscular Endurance, Body Composition, and Back Pain in Firefighters: An Observational Study

**DOI:** 10.3390/muscles2040027

**Published:** 2023-10-24

**Authors:** Shelby Sanregret, Austin Alan Kohler, Andrew Ray Moore, Angelia Maleah Holland-Winkler

**Affiliations:** Department of Kinesiology, Augusta University, Augusta, GA 30909, USA; ssanregret@augusta.edu (S.S.); akohler@augusta.edu (A.A.K.); andmoore@augusta.edu (A.R.M.)

**Keywords:** back pain, firefighters, physical fitness, plank, body fat percentage, core muscular endurance, body mass index, age

## Abstract

Firefighters are at a higher risk for experiencing back pain due to the nature of their job, but physical fitness may help to reduce this risk. Therefore, the primary purpose of this study was to determine if a correlation between subjective back pain severity and core muscular endurance exists in firefighters. A secondary purpose was to determine if age or body composition were correlated with back pain severity. This cross-sectional study was performed at a fire department during their Physical Fitness Assessment Program and included 72 male firefighters. Measures included weight, body fat percentage, maximal plank hold times for core muscular endurance, and the Oswestry Low Back Pain Disability Questionnaire. A Pearson product correlation analysis was performed between back pain score and each of the following variables: body fat percentage, BMI, age, and plank hold times. A second set of correlation coefficient analyses was performed between the same variables exclusively in subjects who reported back pain. This study found that, within this population of firefighters, there was no significant correlation between back pain and plank times or body composition variables, although there was a trend toward significant correlations between BMI and body fat percentage when only subjects with back pain were considered.

## 1. Introduction

Non-specific low back pain is a global concern and significant public health issue, with the lifetime prevalence being reported as high as 84% and a recurrence rate of 24–80% within a year of the first episode [1,2,3]. Non-specific low back pain is defined as pain, muscle tension, or stiffness occurring below the rib margin and above the inferior gluteal folds, sometimes experienced with leg pain and typically confined [3,4,5]. Back pain can have many causes, but certain occupations, especially those that are more physically demanding, may lead to or worsen existing back pain [6]. Firefighting is an occupation that may increase back pain [7].

Low back pain is costly to fire departments, as it is a leading cause of disability in firefighters; fire departments are often left short-staffed while firefighters are recovering from back pain on a workers’ compensation program. Firefighters are at higher risk for back injury due to the strenuous tasks their job requires [7]. In addition, work-related back pain is the main reason firefighters retire early [8,9,10]. While on the job, it may be necessary to perform tasks that may place strain on the back, causing pain or even injury, suffering from back pain may place both the firefighter and victim in danger if the firefighter’s back pain affects their ability to perform certain tasks. Physical fitness can potentially be a way to help prevent this, as it has been shown to increase resistance to low back pain [11]. Increasing resistance to low back pain is especially important in occupations such as firefighting, not only to increase job performance, but also prevent injury [12]. 

Physical fitness is defined as a “set of attributes or characteristics individuals have or achieve that relate to their ability to perform physical activity and activities of daily living” [13,14]. There are five health-related components of physical fitness, which include muscular strength, muscular endurance, cardiorespiratory endurance, body composition, and musculoskeletal flexibility [15,16]. This is especially important in an occupation such as firefighting. Prior to employment or entry into fire school, firefighters are typically required to pass tests to show they are physically able to handle firefighting tasks [17]. However, not all firefighters maintain the physical fitness required for maximal job performance [18]. This decline in physical fitness has the potential to put those firefighters at risk for experiencing health issues such as back pain.

Muscular strength and endurance of the core may play a major role in low back pain [19,20]. Lower levels of core muscular endurance place firefighters at a greater risk of low back pain and are predictive of subsequent work disability [21,22]. The muscles that support the spine have been associated low back pain, with the most common muscles including the iliopsoas, quadratus lumborum, the gluteal muscles, and the multifidus muscles [19,23,24]. Traditional plank exercises have been known to increase core muscular strength and endurance while promoting proper posture [25]. The primary muscles activated during a plank are the rectus abdominis, transverse abdominis, external obliques, internal obliques, erector spinae, multifidus, quadratus lumborum, latissimus dorsi, rhomboids, trapezius, iliopsoas, and the gluteal muscles [26,27]. Core muscular endurance, measured by a plank hold, may be an indicator of risk for back pain, as the muscles typically associated with low back pain are also activated in a plank. 

Previous studies have examined the associations between core strength and back pain in firefighters. Weak back and core muscles have been correlated with low back pain among both firefighters and the general population, with low levels of back muscular endurance being more common among firefighters with a history of low back pain [21,22]. Exercise programs that focus on the back and core muscles have been effective ways of increasing firefighters’ back and core muscular endurance, but the effect of this increased endurance on back injury prevention is still uncertain [28]. In addition, it has been shown that individuals with low back pain have deficits in components of physical fitness; however, the role that muscular strength and endurance plays on the severity of low back pain is still uncertain [29,30]. Because of this, more research is needed to determine the correlation between core muscular endurance and back pain in firefighters. Therefore, the primary purpose of this study was to determine if a correlation between subjective back pain severity and core muscular endurance exists in firefighters. A secondary purpose was to determine if age or body composition were correlated with back pain severity. 

## 2. Materials and Methods

### 2.1. Experimental Design

This cross-sectional study was performed at the local fire department during their Physical Fitness Assessment Program. Age, sex, years of service as a firefighter, reported height, weight, maximal plank hold times, and body composition profile were recorded. Each participant also completed the Oswestry Low Back Pain Disability Questionnaire, which was then scored to measure subjective back pain severity. This study was approved by Augusta University’s Institutional Review Board (IRBnet#1806526-7).

### 2.2. Participants

The participants included 72 males between the ages of 19 and 58 that were firefighters employed by the local Fire Department. All firefighters were required by the Fire Department to complete the Physical Fitness Assessment Program, however, they were not required to volunteer and participate in this study by allowing their data to be shared. Therefore, only the firefighters that voluntarily signed the study’s approved informed consent with a study investigator were included in this study. The participant characteristics are presented in Table 1.

### 2.3. Protocol

Upon arrival in the morning, blood pressure was taken as a safety precaution to continue with the other tests associated with the Physical Fitness Assessment Program. If blood pressure was not abnormal, body composition was then measured via weight and a bioelectrical impedance analysis device (InBody 570, Cerritos, CA, USA), which provided body fat percentage, total lean mass, total fat mass, trunk fat mass, trunk fat percentage (the ratio of fat to lean mass in the trunk), and total body water. The participants were not required to fast for the body composition measures due to the exercise portions of the Physical Fitness Assessment Program.

**Figure 1:**
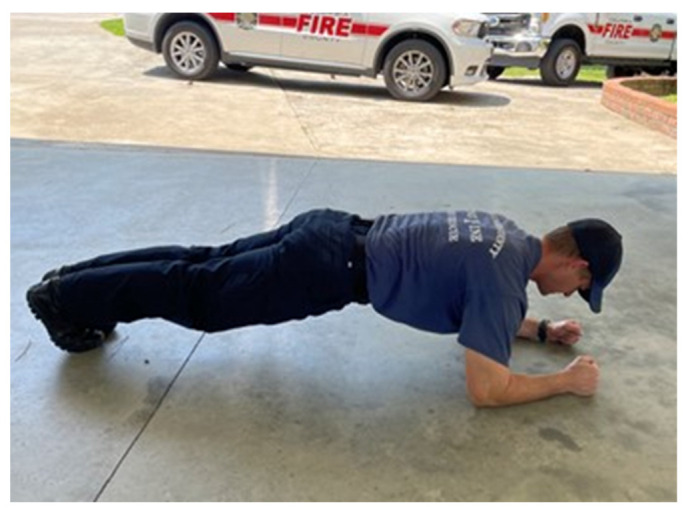
Firefighter in plank position.

The participants were instructed to hold a plank for as long as possible without breaking form. The plank form consists of being in a push-up position but with the arms bent so that the elbows, forearms, and hands rest on the ground, while the elbows are positioned directly below the shoulders. The body should stay in a straight line from the head down to the heels of the feet, as demonstrated in Figure 1. Once the form was broken (i.e., the hips raising or dropping out of the straight line), the plank timer (measured in seconds) was stopped. 

Lastly, the participants filled out the Oswestry Low Back Pain Disability Questionnaire, which serves as a valid and reliable subjective indicator of back pain severity [31]. The questionnaire was filled out with paper and pen and took less than 5 min to complete. Seven components that related to the firefighting occupation regarding back pain were assessed with the questionnaire, including: back pain intensity and pain level when travelling, lifting, walking, sitting, standing, and sleeping. The questionnaire was scored after testing by an investigator for research purposes only and not as a medical diagnosis.

### 2.4. Statistical Analysis

SPSS version 28 was used for all the statistical analyses. The predetermined alpha level for determining statistical significance was 0.05 in all cases. A Pearson product-moment correlation coefficient analysis was performed between back pain score and each of the following variables: age, years of service as a firefighter, BMI, plank hold times, body fat percentage, total lean mass, total fat mass, trunk fat mass, trunk fat percentage, and total body water. The assumption of linearity was met by plotting the back pain score against each variable of interest and visually inspecting the resulting graphs. The collected data were screened for univariate outliers before the analysis. Any data point that was >3.5 standard deviation units away from the group mean for that variable was removed prior to the analysis. One such outlier was observed for the variable plank time, and that data point was removed from the analysis. Subjects who had a missing data point or a removed outlier for any of the variables analyzed were removed from the analyses involving that variable via pairwise deletion. For this reason, different sample sizes are presented for some of the correlation coefficients. The Shapiro–Wilk test was used to assess normality within each variable. Correlation coefficient (*r*) magnitude was interpreted according to the following benchmarks: 0.00–0.10 (negligible), 0.10–0.39 (weak), 0.40–0.69 (moderate), 0.70–0.89 (strong), and 0.90–1.00 (very strong). A negative r indicates an inverse relationship and a positive *r* indicates a direct relationship [32].

## 3. Results

This study had a total of 72 participants who were all males. Only 70 completed the back pain questionnaire, 40 had back pain scores and plank hold time data, and 25 reported back pain scores above zero and plank hold time data. Descriptive statistics are listed in Table 1. The variables of back pain, age, years of service, plank hold time, fat mass, and trunk fat percent were non-normal according to the Shapiro–Wilk test (*p* < 0.05). However, no data transformation or alternate analysis was used, since the Pearson product-moment correlation is robust to normality violations [33,34]. There was no significant relationship between back pain and age (*r* = 0.076, *p* = 0.569, and *n* = 59)*,* years of service (*r* = −0.010, *p* = 0.936, and *n* = 63), BMI (*r* = 0.090, *p* = 0.500, and *n* = 58)*,* body fat percentage (*r* = 0.197, *p* = 0.136, and *n* = 59)*,* or plank time (*r* = −0.268, *p* = 0.094, and *n* = 40), all of which were weak or negligible. There was no significant relationship between back pain and the following body composition variables: lean mass (*r* = 0.097, *p* = 0.463, and *n* = 59), fat mass (*r* = 0.060, *p* = 0.652, and *n* = 58), trunk fat mass (*r* = 0.137, *p* = 0.302, and *n* = 59), trunk fat percent (*r* = −0.027, *p* = 0.840,and *n* = 59), or total body water (*r* = 0.120, *p* = 0.302, and *n* = 59), all of which were weak or negligible. 

### Post Hoc Analyses

It was noted that most of the subjects in this study reported a back pain score of 0 (no back pain), which limited the ability of the correlation analyses to detect linear relationships among all subjects. We decided to perform all the correlation analyses a second time, using only the subjects who reported back pain (a score > 0). The results from these analyses, therefore, describe the relationships between low back pain severity and the selected health and fitness variables in the firefighters who experienced low back pain, specifically. No outliers were detected. The assumption of normality was violated in the variables of back pain and fat mass. 

**Figure 2:**
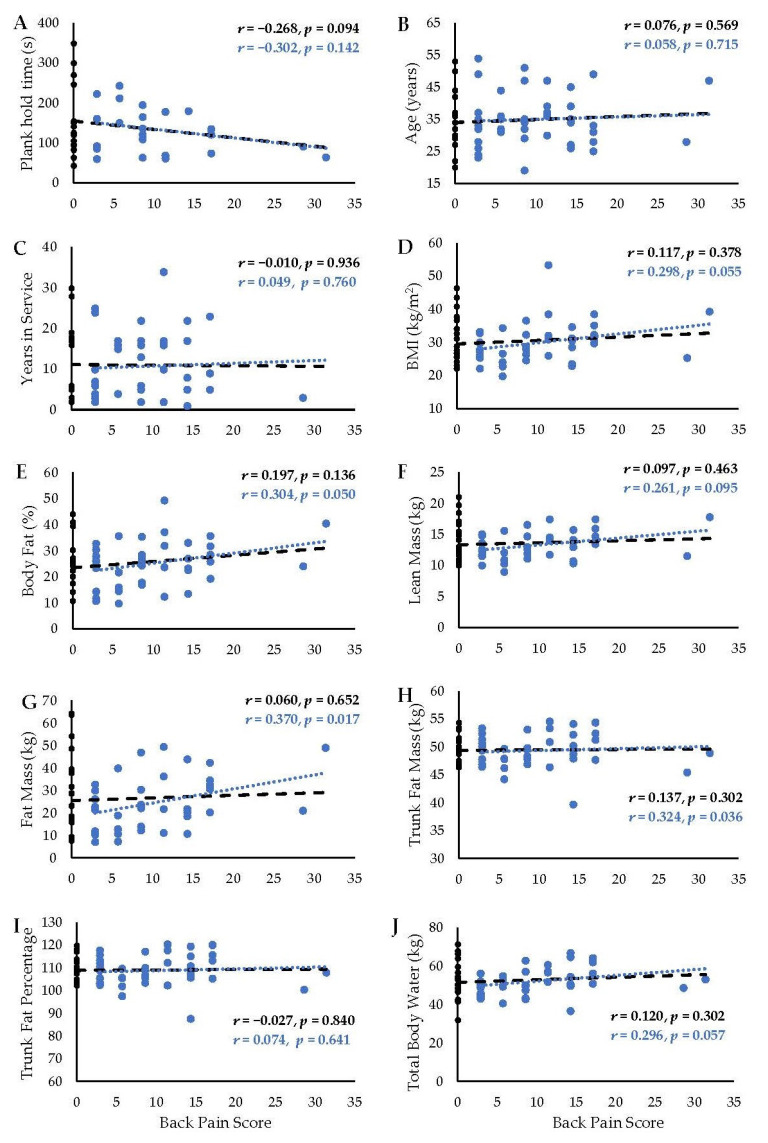
Scatterplots and trendlines representing correlation between back pain score and (**A**) plank hold time, (**B**) age, (**C**) years of service as a firefighter, (**D**) BMI (body mass index), (**E**) body fat percentage, (**F**) total lean mass, (**G**) total fat mass, (**H**) trunk fat mass, (**I**) trunk fat percentage, and (**J**) total body water. Correlation coefficients (*r*) and exact *p*-values are presented. Black text and trendline indicate results for all subjects (black and blue markers); blue text and trendline indicate data only for subjects reporting back pain (blue markers only).

There was a significant correlation between back pain and the variables of fat mass (*r* = 0.370, *p* = 0.017, and *n* = 41) and trunk fat mass (*r* = 0.324, *p* = 0.036, and *n* = 42). There was a trend toward a significant correlation between back pain and the variables of BMI (*r* = 0.298, *p* = 0.055, and *n* = 41) and body fat percentage (*r* = 0.304, *p* = 0.050, and *n* = 42). There was no significant correlation between low back pain and the variables of age (*r* = 0.058, *p* = 0.715, and *n* = 42), years of service (*r* = 0.049, *p* = 0.760, and *n* = 42), plank time (*r* = −0.302, *p* = 0.142, and *n* = 25), lean mass (*r* = 0.261, *p* = 0.095, and *n* = 42), trunk fat percentage (*r* = 0.074, *p* = 0.641, and *n* = 42), or total body water (*r* = 0.296, *p* = 0.057, and *n* = 42). All the correlations were considered to be weak or negligible. The results from all the correlation analyses are represented visually in Figure 2.

## 4. Discussion

As back pain is a major contributor to firefighter disability and even early retirement, it is important to continue to explore the primary contributors to back pain in this population. This study assessed core muscular endurance via a plank hold, body composition profile via a bioelectrical impedance analysis, age, and years of service as a firefighter as possible predictors associated with back pain severity. The main finding was that fat mass and trunk fat mass were significantly correlated with back pain severity. Firefighters in the United States have been commonly categorized at an average physical fitness level, with many being overweight or obese [35]. The physically demanding nature of the firefighting occupation may place firefighters at an increased risk of injury if their physical fitness level does not meet the level demanded by their occupational tasks. Similar to the above-mentioned findings in the United States, the average BMI in our sample of firefighters was 30.28, which is categorized as obese [36]. The average body fat percentage was 25.18%, which, for the age range of the participants in this study, can be categorized from a poor to average fitness for men [37]. Interestingly, the average plank score was 143 s, which is ranked between the 70th and 80th percentile for 20-year-old men and therefore categorized as a high level of abdominal endurance [38]. The average back pain score was 6.6, which is interpreted as minimal disability. This value was lower than anticipated due to many participants reporting a score of zero on their Oswestry Low Back Pain Questionnaire. Because the reported back pain scores were lower than expected, secondary analyses were run using only the data from the participants that reported back pain scores above zero. This serves as a limitation to this study, as it reduced the participant sample size used in the statistical analysis.

In the participants who reported experiencing back pain, fat mass and trunk fat mass were significantly correlated with back pain. This finding may be expected, because an increase in fat translates to an increase in the stress associated with postural stability that is essential for meeting the occupational demands of firefighters. However, it is important to note that both subgroups analyzed (firefighters with and without back pain) had nearly equivalent levels of fat mass and trunk fat mass, and were not significantly different according to independent *t*-tests. This suggests that whatever the precise cause of back pain is, an increase in absolute body fat may exacerbate the experienced severity. 

Previous research has been conducted to help determine the predictors of back pain in firefighters, as well as how core muscular strength and endurance can affect job performance. Damrongsak et al. (2018) found that variables including occupational stress, age, history of back pain, and BMI were good predictors of the probability of current back pain in firefighters, but the variables of perceived supervisor support, job satisfaction, and physical fitness did not contribute to the prediction of back pain [12]. We examined more specific measures of body composition in addition to BMI, and found that these variables more closely corresponded to back pain severity. Future studies and tactical athlete practitioners should analyze specific indices of body composition, when feasible, rather than relying on BMI, which cannot discern between fat and muscle mass. Moreover, Moon et al. (2015) suggested that increasing the muscular strength and endurance of the lumbar and abdominal region may reduce back pain via proper posture and reductions in spinal loading [39]. We did not assess the causal nature of core muscle endurance on back pain in this correlational study, but we did find a weak negative relationship between plank time and back pain that was limited by the small sample size. This observation is consistent with the finding that excess weight may hinder core muscular endurance as a negative relationship between obesity and maximum plank hold time [40]. That is, the plank hold time and absolute fat measures may be assessing similar characteristics in different ways. The relationship between core muscular endurance and body composition with back pain is controversial, as studies have found conflicting results [41]. For instance, a 12-week exercise program in firefighters improved their body composition, cardiorespiratory fitness, muscular strength, power, endurance, and flexibility, however, it did not affect occupational low-back loading measures, which is predictive of an unchanged risk of low back injury [42]. On the other hand, a randomized controlled 12-month trial that consisted of back and core exercises, delivered either via telehealth or on-site supervision two times per week, resulted in reduced lost work time related to low back pain in the firefighters in both the telehealth and on-sight supervision groups compared to a control group [43]. Therefore, it is important to continue to explore this topic to better understand the factors that contribute to back pain and how to prevent it in this population. Due to its multifaceted etiology, a multi-pronged intervention may be required to combat this condition in firefighters (i.e., physical activity to improve postural stability paired with nutritional counselling to improve body composition indices). For instance, a multifaceted back injury prevention education program called the Back Informed Program was implemented in firefighters and included on-site job-specific education, ergonomic, exercise, and pain management advice, and hands-on practice sessions. There was a significant reduction in lost work time after both year one and year two compared to the year before the program was implemented [44]. Developing research-based physical fitness and educational programs to reduce back pain is essential to reducing the fear and anxiety in those experiencing back pain, as these psychological factors have been shown to intensify and continue feelings of pain [45]. 

## 5. Conclusions

Our study did not support a correlation between core muscular endurance and back pain, possibly due to its small sample size and population with a high level of core muscular endurance and low levels of back pain. Fat mass and trunk fat mass were significantly correlated with back pain severity, suggesting that interventions which reduce body fat may be the most promising when investigating strategies for reducing back pain incidence and severity. 

## Figures and Tables

**Table 1:** Subject demographic information and descriptive statistics.

	All Subjects		Subjects with Back Pain		Subjects without Back Pain	
	M	SD	M	SD	M	SD
Age (years)	34.59	(8.56)	34.92	(8.27)	33.76	(9.46)
Height (cm)	173.17	(4.42)	179.69	(7.38)	179.00	(8.84)
Weight (kg)	96.00	(23.40)	94.31	(20.92)	101.00	(30.21)
BMI (kg/m^2^)	30.28	(6.39)	29.87	(5.91)	31.30	(7.52)
Years of service	11.01	(8.39)	10.67	(8.08)	11.67	(9.1)
Back Pain Score	6.63	(7.10)	9.95	(6.52)	0	(0)
Plank Hold Time (s)	142.85	(70.03)	132.48	(55.14)	160.13	(89.10)
Body Fat%	25.18	(8.82)	25.21	(8.55)	25.10	(9.72)
Lean Mass (kg)	19.10	(2.91)	18.96	(2.53)	19.47	(3.75)
Fat Mass (kg)	26.33	(13.80)	24.37	(11.43)	31.09	(17.83)
Trunk Fat Mass (kg)	32.40	(4.96)	32.18	(4.5)	32.95	(6.52)
Trunk Fat%	109.27	(6.66)	108.77	(6.80)	110.54	(6.34)
Total Body Water (kg)	52.47	(7.98)	52.10	(6.91)	53.42	(10.35)

## Data Availability

The data presented in this study are openly available in Open Science Framework (OSF) at the web page https://osf.io/vzpb6/ (accessed on 29 June 2023) with the DOI 10.17605/OSF.IO/VZPB6.
